# Supplementary material for: Co-Expression of Multiple PAX Genes in Renal Cell Carcinoma (RCC) and Correlation of High PAX Expression with Favorable Clinical Outcome in RCC Patients
Source: Int J Mol Sci. 2023 Jul 14;24(14):11432. doi: 10.3390/ijms241411432 (PMC10380508; doi:10.3390/ijms241411432)
Supplement: Supplementary file 1 [file ijms-24-11432-s001.zip › ijms-2445052-supplementary.pdf]

Co-Expression of Multiple *PAX* Genes in Renal Cell Carcinoma (RCC), and Correlation of High *PAX* Expression with Favorable Clinical Outcome in RCC Patients

Lei Li, Caiyun G. Li, Suzan N. Almomani, Sultana Mehbuba Hossain and Michael R. Eccles

Supplementary Figures:

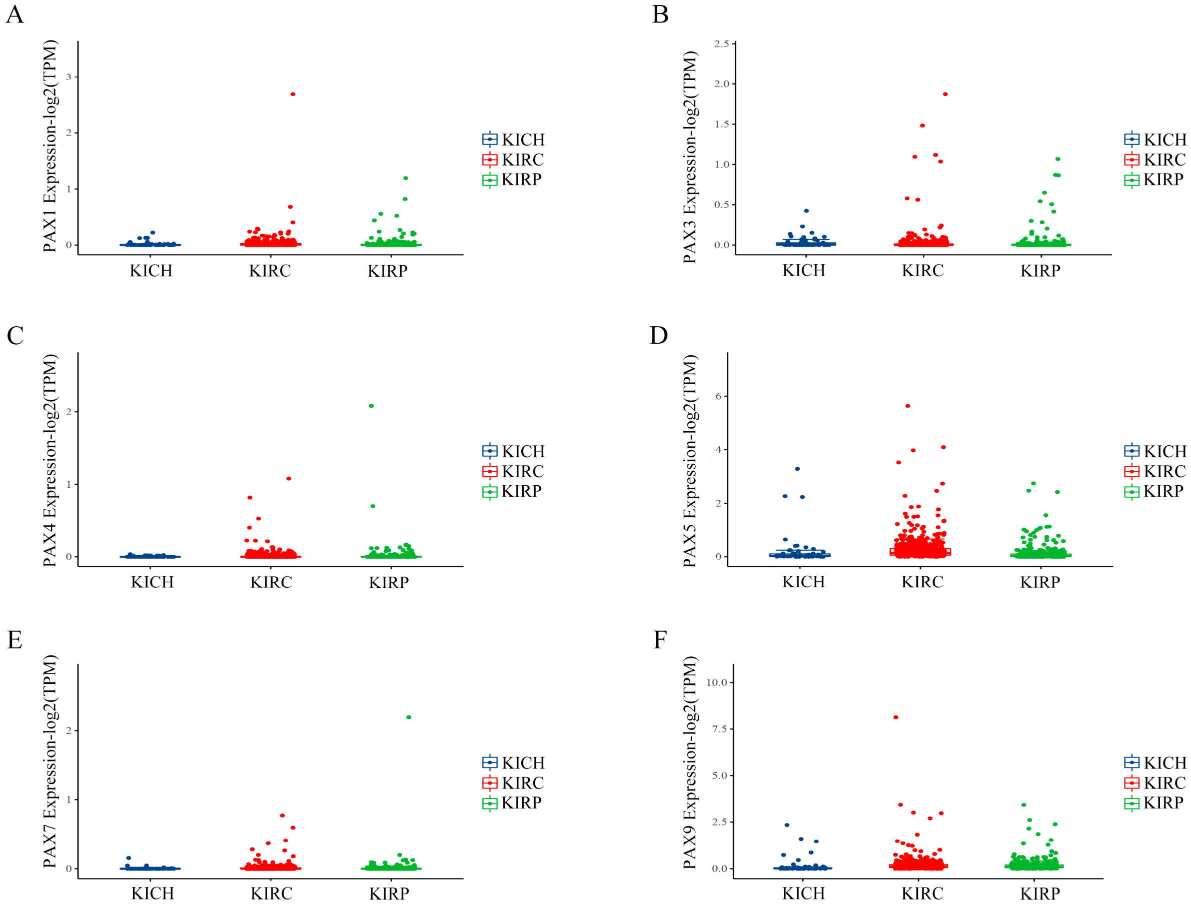

Supplementary Figure S1. Analysis of the expression level of (A) *PAX1*, (B) *PAX3*, (C) *PAX4*, (D) *PAX5*, (E) *PAX7*, (F) *PAX9*, in KICH, KIRC, and KIRP.

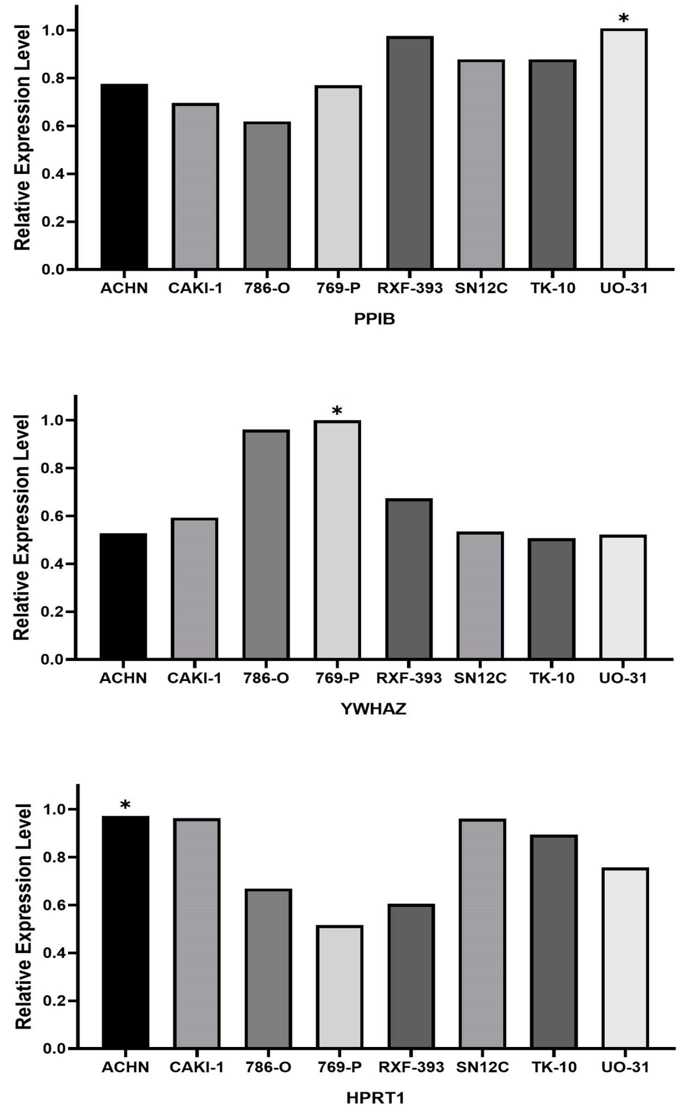

Supplementary Figure S2. Reference gene expression profiles in human cell lines. The *PPIB*, *YWHAZ*, and *HPRT1* gene expression profiles were determined in RCC cell lines. Expression data of reference genes are presented relative to the "calibrator" (the highest expressing cell lines, *).

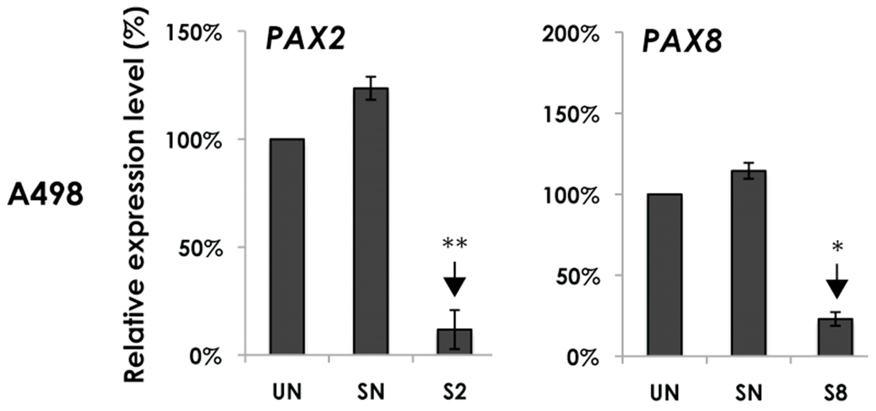

Supplementary Figure S3. *PAX2* and *PAX8* were successfully knocked down in A498 cells. A498 cells were treated with the indicated siRNAs (x-axis). Transcript levels were determined at 72 h post-siRNA treatments. *PAX* expression was normalized to the respective reference genes, and plotted relative to the UN sample (= 100%). Arrows indicate the *PAX* knockdowns by the respective siRNA. Statistical analysis was performed in comparison to the SN treated samples. *, $P < 0.05$; **, $P < 0.01$; and ***, $P < 0.001$ (one-way ANOVA, Tukey's test).

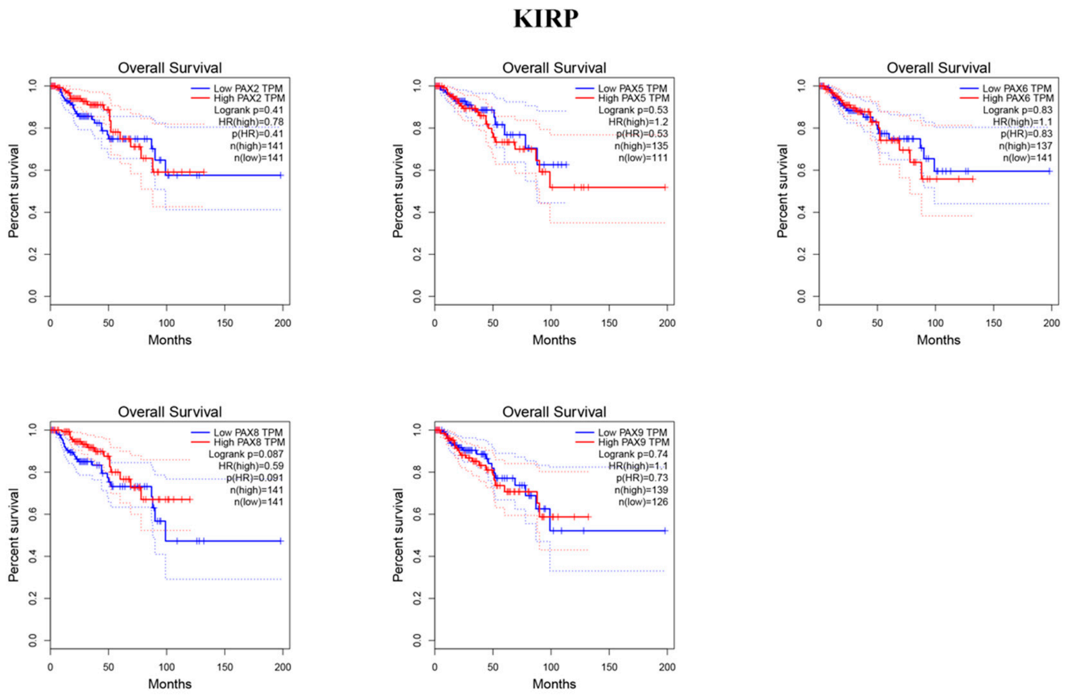

Supplementary Figure S4. The Kaplan-Meier curves show the relationship between

the *PAX* gene family and overall survival of RCC patients (KIRP). For some genes, curve maps cannot be drawn due to insufficient samples or gene expression levels.

KIRC

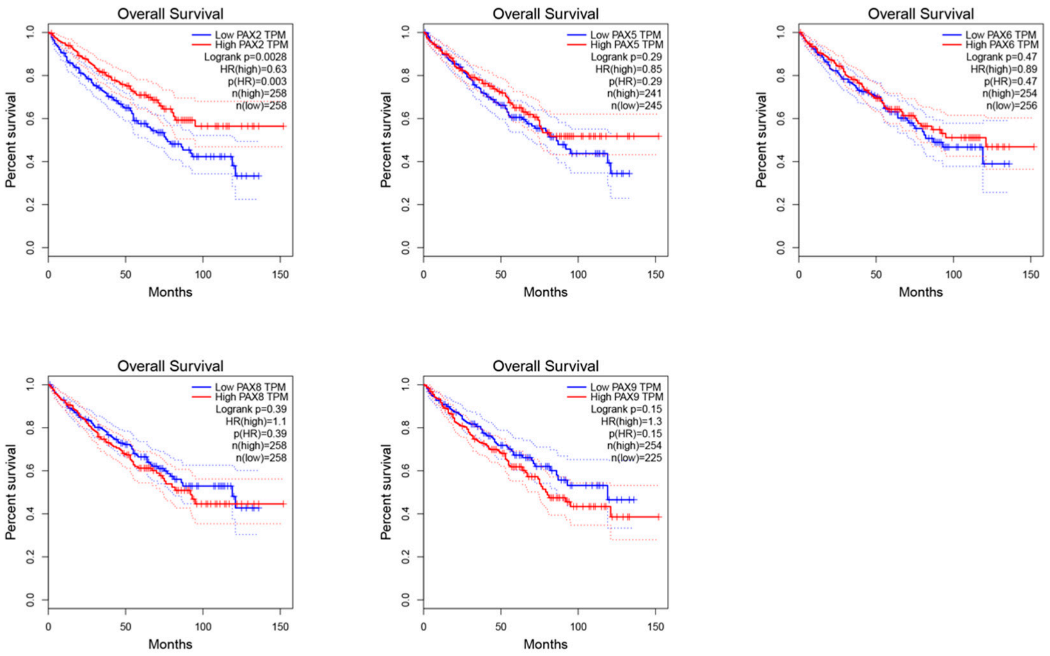

Supplementary Figure S5. The Kaplan-Meier curves show the relationship between the *PAX* gene family and overall survival of RCC patients (KIRC). For some genes, curve maps cannot be drawn due to insufficient samples or gene expression levels.

KICH

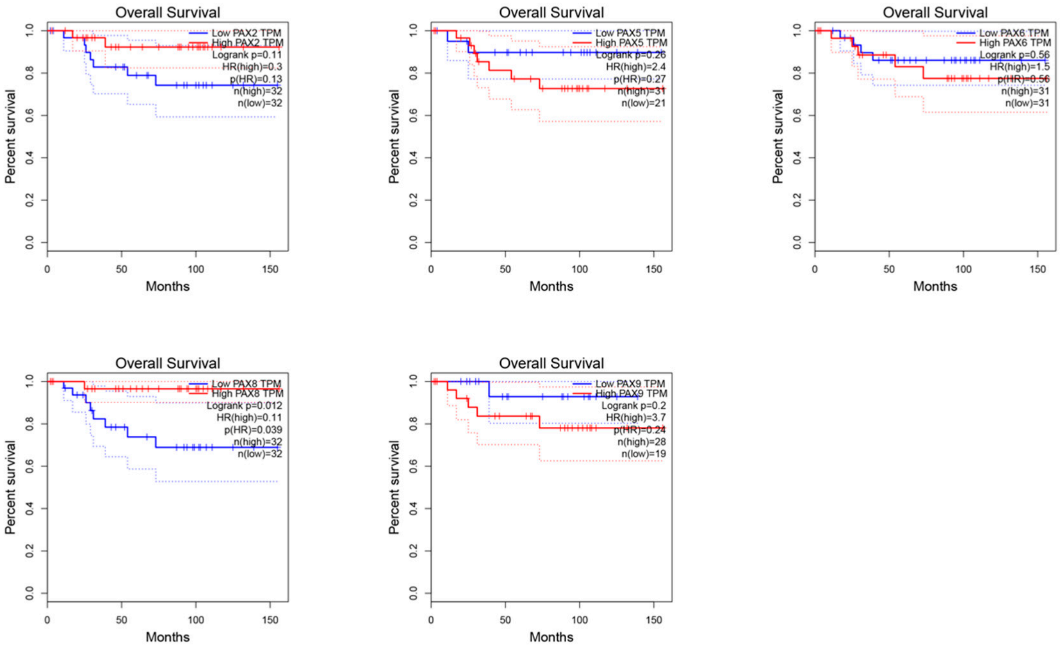

Supplementary Figure S6. The Kaplan-Meier curves show the relationship between the *PAX* gene family and overall survival of RCC patients (KICH). For some genes, curve maps cannot be drawn due to insufficient samples or gene expression levels.

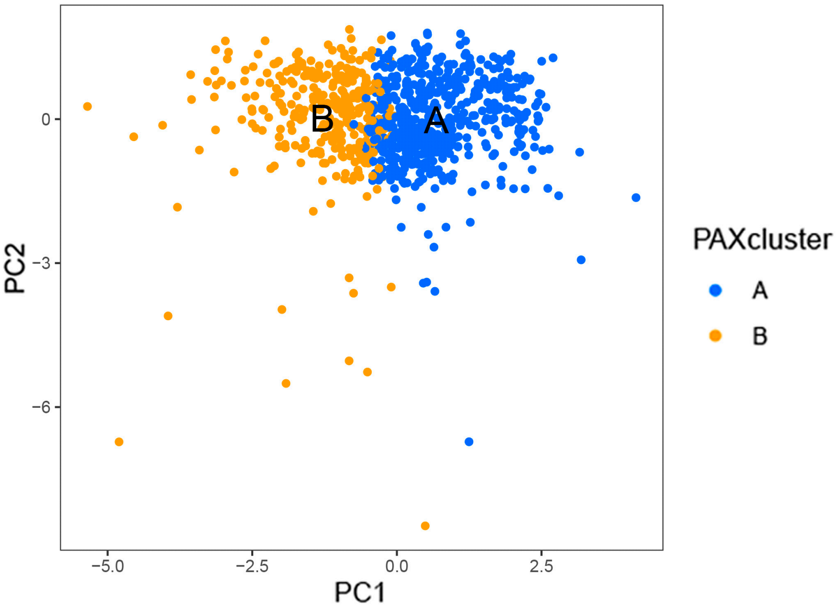

Supplementary Figure S7. Principal component analysis (PCA) analysis of PAXclusterA and PAXClusterB.

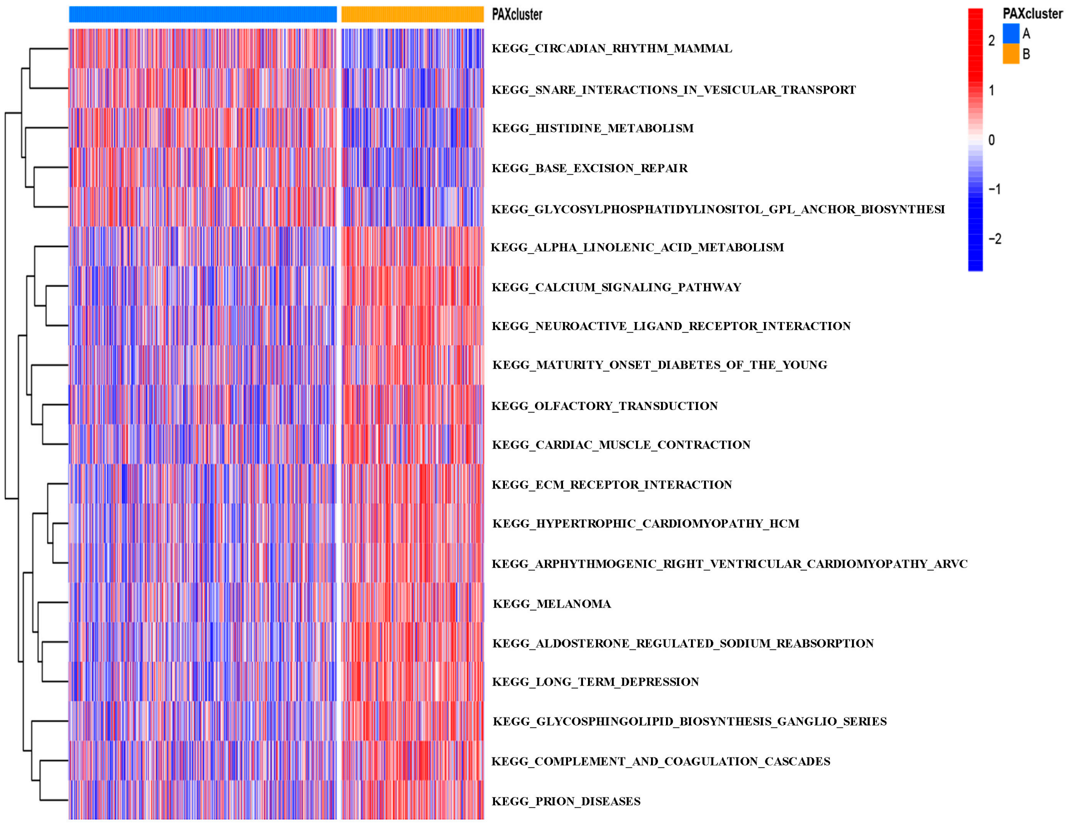

Supplementary Figure S8. GSVA enrichment analysis shows the activation states of biological pathways in the PAXClusterA and PAXClusterB subtypes. The heatmap was used to visualize these biological processes. Red represented activated pathways and blue represented inhibited pathways.

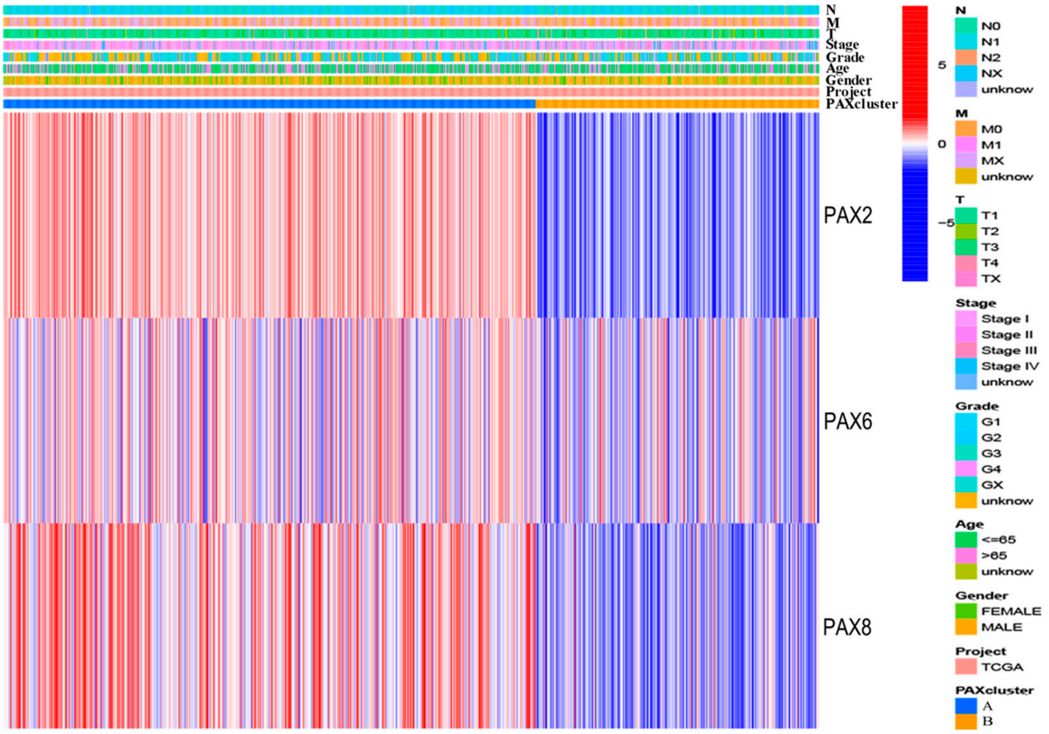

Supplementary Figure S9. Heatmap of clinical features between PAXClusterA and PAXClusterB.

Supplementary Tables:

Supplementary Table S1. Mammalian cell lines used in this study.

Cell type	Cell line	Culture medium *	Remarks
Renal cell carcinoma (RCC)	A498<br>ACHN<br>CAKI-1<br>769-P<br>786-O<br>SN12C<br>TK-10<br>UO-31<br>RXF-393	RPMI-1640 (10% FBS)	All RCC cell lines were derived from clear cell RCC, except for ACHN (papillary RCC).

All cell lines (unless otherwise stated) were obtained from either the American Type Culture Collection (Virginia, USA) or the National Cancer Institute Division of Cancer Treatment and Diagnosis tumour repository (Maryland, USA).

* All cell culture media and supplements were purchased commercially.

Supplementary Table S2. Housekeeping genes primers used in this study.

Housekeeping genes	Forward primer	Reverse primer
PPIB	ATGATCCAGGGCGGAGACTT	CAGGCCCGTAGTGCTTCAG
YWHAZ	ACTTGACATTGTGGACATCGGATAC	GTTGGAAGGCCGGTTAATTTTC
HPRT1	ATTATGGACAGGACTGAACGTCTTG	TGAGCACACAGAGGGCTACAAT

Supplementary Table S3. siRNAs used in this study.

siRNA target	Name (Abbreviation)	Sequence (5'->3')	Manufacturer (siRNA ID) *
Non-target	SMARTpool ON-TARGETplus siCONTROL non-targeting pool (SN)	N/A	Dharmacon (D-001810-10)
PAX2	SMARTpool ON-TARGETplus siPAX2 (S2)	GAAGUCAAGUCGAGUCUAUUU<br>CGACAGAACCCGACUAUGUU<br>GGACAAGAUUGCUGAAUACUU<br>CAUCAGAGCACAUCAAAUCUU	Dharmacon<br>(L-003921-00)
PAX6	SMARTpool ON-TARGETplus siPAX6 (S6)	CCAAGCGUGUCAUCAAUAA<br>GGCAAUCGGUGGUAGUAAA<br>GUGCGACAUUUCCCGAAUU<br>AACCUGAUAUGUCUCAAUA	Dharmacon<br>(L-011098-00)
PAX8	SMARTpool ON-TARGETplus siPAX8 (S8)	CAUCCGGCCUGGAGUGAUA<br>CCUCACAACUCCAUCAGAU<br>CGACAUCUCUCGCCAGCUC	Dharmacon<br>(L-003778-00)

		CAGGAUAGCUGCCGAC UAA	
PAX8	Silencer Pre-designed siPAX8 (A8)	GCAUUGACUCACAGAG CAGTT	Ambion (114354)

N/A: not available.

* Both Dharmacon and Ambion are from USA.

Supplementary Table S4. Antibodies used in this study.

Primary antibody	Catalogue no. (manufacturer)	Dilution	Secondary antibody*	Dilution
PAX2	71-6000 (Invitrogen, USA)	1:500	Anti-rabbit IgG	1:2000
PAX8	PA 030 (Biopat, Italy)	1:20,000	Anti-rabbit IgG	1:20,000
β-actin	ab6276 (Abcam)	1:5000	Anti-mouse IgG	1:5000

β-actin antibody was provided by Prof. A. W. Braithwaite (University of Otago, NZ).

* All secondary antibodies were labelled with horseradish peroxidase (HRP) and were purchased from Sigma- Aldrich (USA): anti-rabbit IgG (A0545), anti-mouse IgG (A9917).
